# The Extension of the LeiCNS-PK3.0 Model in Combination with the “Handshake” Approach to Understand Brain Tumor Pathophysiology

**DOI:** 10.1007/s11095-021-03154-1

**Published:** 2022-03-07

**Authors:** Makoto Hirasawa, Mohammed A. A. Saleh, Elizabeth C. M. de Lange

**Affiliations:** Division of Systems Biomedicine and Pharmacology, Leiden Academic Centre for Drug Research, Leiden University, Einsteinweg 55, 2333 CC Leiden, The Netherlands

**Keywords:** blood-tumor barrier, brain tumors, physiologically based pharmacokinetic model, tumor pathophysiology

## Abstract

Micrometastatic brain tumor cells, which cause recurrence of malignant brain tumors, are often protected by the intact blood–brain barrier (BBB). Therefore, it is essential to deliver effective drugs across not only the disrupted blood-tumor barrier (BTB) but also the intact BBB to effectively treat malignant brain tumors. Our aim is to predict pharmacokinetic (PK) profiles in brain tumor regions with the disrupted BTB and the intact BBB to support the successful drug development for malignant brain tumors. LeiCNS-PK3.0, a comprehensive central nervous system (CNS) physiologically based pharmacokinetic (PBPK) model, was extended to incorporate brain tumor compartments. Most pathophysiological parameters of brain tumors were obtained from literature and two missing parameters of the BTB, paracellular pore size and expression level of active transporters, were estimated by fitting existing data, like a “handshake”. Simultaneous predictions were made for PK profiles in extracellular fluids (ECF) of brain tumors and normal-appearing brain and validated on existing data for six small molecule anticancer drugs. The LeiCNS-tumor model predicted ECF PK profiles in brain tumor as well as normal-appearing brain in rat brain tumor models and high-grade glioma patients within twofold error for most data points, in combination with estimated paracellular pore size of the BTB and active efflux clearance at the BTB. Our model demonstrated a potential to predict PK profiles of small molecule drugs in brain tumors, for which quantitative information on pathophysiological alterations is available, and contribute to the efficient and successful drug development for malignant brain tumors.

**Supplementary Information:**

The online version contains supplementary material available at 10.1007/s11095-021-03154-1.

## Introduction

Glioblastomas (GBM) are the most common and most malignant brain tumors, with a 5-year relative survival of only 6.8% (1). In spite of many efforts to develop novel therapeutic agents, only 4 drugs (temozolomide, lomustine, carmustine and bevacizumab) and 1 device (tumor treatment fields) are currently available for the treatment of high-grade gliomas (HGG) including GBM (2), and the prognosis remains poor with inevitable tumor recurrence (3). One of the major causes of treatment failure is attributable to the insufficient drug exposure to tumor tissue, due to the presence of the blood–brain barrier (BBB) (3). The BBB is composed of specialized brain microvascular endothelial cells (BMEC) surrounded by pericytes, basement membrane and astrocyte end-feet, and strictly limits the delivery of drugs from blood into brain parenchyma as well as regulates homeostasis of the central nervous system (CNS) (4).

Malignant brain tumors are known to disrupt the integrity of the BBB and widen the inter-endothelial tight junctions during diseases progression, which is referred to as the blood-tumor barrier (BTB) (5, 6). The intratumoral microvasculature (MV) with the disrupted BTB, especially in HGG, are believed to be generally leakier than healthy brain MV with the intact BBB, as demonstrated by the intratumoral accumulation of brain impenetrant contrast agents in essentially all GBM (7). However, clinical evidence demonstrated that there is also a clinically significant tumor burden with an intact BBB in all GBM (3, 8, 9). Several preclinical studies have also shown that the BTB displays highly heterogeneous permeability not only in animal glioma models but also in animal brain metastasis models (10–12). These findings indicate that it is essential to deliver pharmacologically active drugs across not only the disrupted BTB but also the intact BBB to effectively treat HGG, while many drugs with poor BBB penetration have been tested and failed in clinical trials (6). Therefore, the prediction of pharmacokinetic (PK) profiles in tumor tissues in both regions is crucially important for the successful drug development for HGG. In addition, since novel drug candidates are selected using experimental animal brain tumor models during the drug discovery process, it is of great significance to quantitatively understand and describe the impact of pathophysiological alterations in each animal model on tumor drug exposure for an appropriate understanding of PK-pharmacodynamics (PD) relationships and the selection of right candidates for clinical trials which are likely to reach the target site at therapeutic levels to show the desired pharmacological responses.

Our group recently developed a comprehensive physiologically based pharmacokinetic (PBPK) model, “LeiCNS-PK3.0”, for the prediction of CNS PK profiles of small molecule drugs with a wide range of physicochemical properties and demonstrated the drug dependent impact of altered physiological conditions on unbound PK profiles in multiple physiological CNS compartments, including brain extracellular fluid (ECF), intracellular fluid (ICF) and cerebrospinal fluid (CSF) (13, 14).

In the present study, we extended the LeiCNS-PK3.0 model to predict unbound PK profiles in brain tumor tissue simultaneously with those in normal-appearing brain tissue, by adding brain tumor compartments and integrating pathophysiological properties of brain tumors. Some of the pathophysiological parameters of brain tumors are, however, currently unavailable for the “bottom-up” model development, in spite of the urgent demand for the development of novel therapeutic agents for malignant brain tumors. In this study, we used the “handshake” approach, i.e., fitting existing data, to understand and estimate what pathophysiological alterations could be in brain tumors, making the most of existing data. In addition, tumor heterogeneity, especially in terms of heterogenous barrier functions of the BBB/BTB within and between tumor masses, ideally needs to be addressed in predicting drug exposure to tumor tissue. Nevertheless, firstly we used a categorical approach focusing on tumor core region and normal-appearing brain region in the present study, based on the availability of quantitative information on pathophysiological alterations.

## Materials and Methods

### *In Vivo* Unbound PK Profiles

*In vivo* PK data of six small molecule drugs including methotrexate, temozolomide, ganciclovir, gemcitabine, letrozole and cisplatin was obtained by an extensive literature search using the National Library of Medicine PubMed database with free text terms “brain tumor” OR “glioma” AND “microdialysis”, considering synonyms. These drugs were selected as their plasma PK data and microdialysis (MD) data in both tumor tissue and control (healthy, sham, contralateral or non-contrast-enhancing (NE)) brain tissue in rat brain tumor models or human patients are available in the same research article. In order to appropriately understand the impact of pathophysiological alterations in brain tumors on tumor PK profiles, which is the major purpose of this study, control brain PK data measured under the same conditions as tumor PK is crucially important as the “best basis” for further analysis on tumor compartments and therefore publications lacking control brain PK data were excluded. No other factors than the availability of PK data were considered in the drug selection. In addition, methotrexate data in C6 glioma model rats reported by Dukic *et al.* (15) was excluded because BBB functionality in the contralateral hemisphere of this model was likely to be already affected by the ipsilateral C6 glioma. Table I includes data references. Total drug concentrations in plasma were corrected using the fraction unbound in plasma where needed. Kp_uu,ECF_, the ratio of the unbound drug concentration in ECF to that in plasma at steady state, was either available from literature or calculated using the reported ratio of area under the unbound concentration–time curve in ECF to that in plasma. It should be noted that PK profile after the administration of cisplatin was reported as that of free platinum (16) and probably includes metabolites.

**Table I:** Summary of *in vivo* PK Data and fup Collected from Literature

Species	Drug	PK data reference	fup		Non-tumor brain		Brain tumor		
			Value	Reference	Brain type	Kp_uu,ECF_	Tumor model	Tumor type	Kp_uu,ECF_
Rat brain tumor models	Methotrexate	(17)	0.448	(19)	Sham brain	0.114	RG-2	Rat glioma	0.105
		(18)			Healthy brain	0.118	R-6	Rat rhabdomyosarcoma	0.250
					Sham brain	0.114			
					Contralateral hemisphere	0.096			
		(15)			Contralateral hemisphere	0.00527	CNS1	Rat glioma	0.123
	Temozolomide	(20)	0.85	(21)	Contralateral hemisphere	0.262	SF188/V +	Human glioma	0.227
	Ganciclovir	(22)	-	-	Contralateral hemisphere	0.269	BT4C	Rat glioma	0.785
	Gemcitabine	(23)	-	-	Healthy brain	0.065	C6	Rat glioma	0.186
					Contralateral hemisphere	0.085			
	Letrozole	(24)	0.38	(24)	Healthy brain	0.786	C6	Rat glioma	1.40
					Contralateral hemisphere	0.678			
	Cisplatin	(16)	-	-	Contralateral hemisphere	0.04	9L	Rat gliosarcoma	0.69
Human brain tumor patients	Methotrexate^a^	(25)	0.677	(26)	Non-contrast-enhancing brain region	0.0473 (C) 0.139 (D)	-	High-grade glioma	0.415 (A) 0.451 (B)

ECF: brain extracellular fluid; fup: fraction unbound in plasma; Kp_uu,ECF_: the ratio of the unbound drug concentration in ECF to that in plasma at steady state

^a^ Parentheses represent patient ID

### Addition of Brain Tumor Compartments to the LeiCNS-PK3.0 Model

**Fig. 1:**
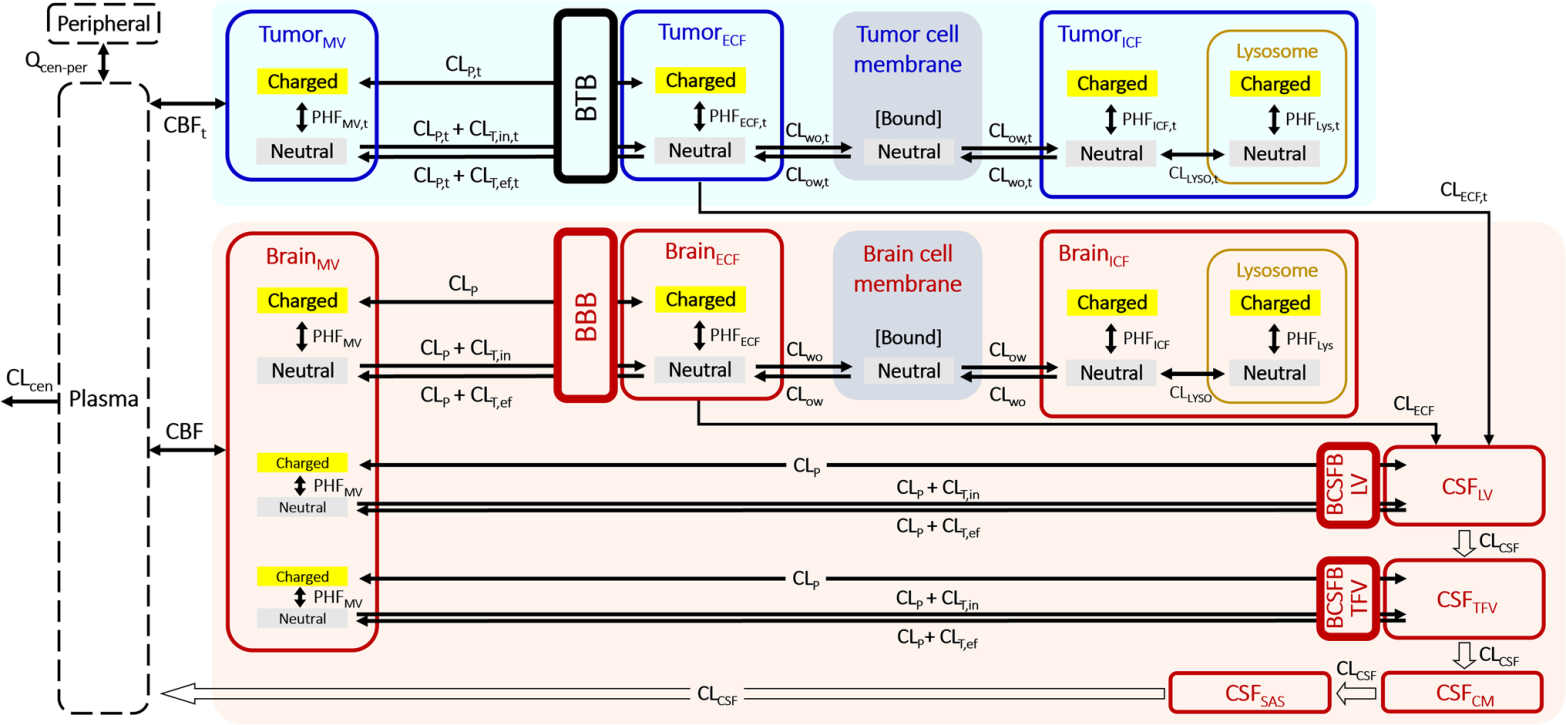
Structure of the LeiCNS-tumor model. Five tumor compartments (highlighted in blue) were added between plasma and CSF_LV_ compartments of the LeiCNS-PK3.0 model. Descriptions with subscript “t” represent processes in brain tumor compartments. [Barriers] BBB: blood brain barrier; BCSFB: blood CSF barrier; BTB: blood tumor barrier; [Compartments] CM: cisterna magna; CSF: cerebrospinal fluid; ECF: brain extracellular fluid; ICF: brain intracellular fluid; LV: lateral ventricles; MV: brain microvasculature; SAS: subarachnoid space; TFV: 3rd & 4th ventricles; [Factors] PHF: pH factor; [Flows] CBF: cerebral blood flow; CL_cen_: central clearance; CL_ECF_: ECF bulk flow; CL_CSF_: CSF flow; CL_LYSO_: transmembrane clearance of lysosomes; CL_ow_: lipid-to-water clearance; CL_wo_: water-to-lipid clearance; CL_p_: paracellular transport clearance; CL_T,ef_: efflux transcellular clearance; CL_T,in_: influx transcellular clearance.

The LeiCNS-PK3.0 model consists of an empirical plasma PK model and a nine-compartment CNS model with the physiological parameters. CNS compartments include brain MV plasma, brain ICF, brain ECF, brain cell membrane and lysosomes, and four CSF compartments (lateral ventricle (LV), 3^rd^ and 4^th^ ventricle (TFV), cisterna magna (CM) and sub-arachnoid space (SAS)). More information on the model building, the physiological parameters of rat and human under healthy condition, and the associated equations can be found at (13, 27). Here, five compartments of brain tumor (MV, ICF, ECF, tumor cell membrane and lysosomes) having the same structure as the healthy brain model were added between plasma and LV compartments. Because the spatial information on tumor site within the whole brain in each individual is not available, no transport between tumor ECF and healthy brain ECF was assumed. Figure 1 displays the model structure of the LeiCNS-PK3.0 model with brain tumor compartments (hereinafter referred to as the “LeiCNS-tumor” model).

### Data Analysis and Software

Parameter estimation was performed using NONMEM version 7.4.3 (ICON, Dublin, Ireland) (28). General data analysis, visualization and LeiCNS-PK3.0 simulations were performed using R version 4.0.3 (29). LeiCNS-PK3.0 simulations were performed using RxODE package version 0.9.2.1 (30), using the LSODA (Livermore Solver for Ordinary Differential Equations) Fortran package. WebPlotDigitizer version 4.4 (https://apps.automeris.io/wpd/) was used to extract data from literature.

### Empirical Plasma PK Models

Plasma PK models of unbound drugs were developed using *in vivo* data with a non-linear mixed effects modeling approach, where one- or two- compartment models were evaluated. For ganciclovir, a lag time was tested considering the delayed onset of absorption after intraperitoneal administration (22). Interindividual variability (IIV) was tested for methotrexate PK data in rats reported by de Lange *et al.* (18) using exponential models for clearance (CL) and volume of distribution of central compartment. Residual unexplained variability (RUV) was included using either proportional or combined proportional/additive error models. The final model was selected based on the objective function value and visual predictive check (VPC) plots to compare the model fit to unbound PK profiles in plasma.

### Drug-Specific Physicochemical Parameters

The physicochemical parameters of six drugs including molecular weight, the ionization constants of the strongest acidic group (pK_a_) and the strongest basic group (pK_b_) that are the most significant parameters determining the proportion of charged and uncharged molecule at a given pH, and lipophilicity (logP_o/w_) were collected from DrugBank version 5.1.7 (31) and are listed in Table II. Experimental logP_o/w_ values were used, while calculated pK_a_/pK_b_ values by the MARVIN method provided by CHEMAXON (32) were used. Parameters of cisplatin were used as representative parameters for all the cisplatin-derived free platinum compounds, possibly including cisplatin and its metabolites, existing in rats after the administration of cisplatin. Asymmetry factors (AF) were calculated from Kp_uu,ECF_ in control brain using the LeiCNS-PK3.0 equations at steady state as previously reported (13).

**Table II:** Drug-Specific Physicochemical Parameters of Six Small Molecule Anticancer Drugs Included in this Study

Drug	Molecular weight	Charge class	Strongest acidic pK_a_	Strongest basic pK_b_	logP_o/w_
Methotrexate	454.45	Acid	3.41	2.81	-1.85
Temozolomide	194.15	Neutral	10.51	-3.6	-1.153
Ganciclovir	255.23	Neutral	10.16	1.76	-1.66
Gemcitabine	263.20	Neutral	11.52	3.65	-1.4
Letrozole	285.30	Neutral	NA	2.17	2.5
Cisplatin	300.05	Neutral	NA	NA	-2.19

NA: not applicable

### Pathophysiological Parameters of Brain Tumors

Quantitative information on pathophysiological alterations in brain tumor tissues, including tumor blood flow, volume fractions of MV and ECF, extracellular and intracellular pH, in experimental animal models as well as human patients was obtained by an extensive literature search using the National Library of Medicine PubMed database with free text terms “brain tumor model name or brain tumor type” AND “parameter name”, considering synonyms. When multiple values were found, the mean value was used for each animal model or tumor type. When no information was found for a certain animal model or tumor type, the mean value of the same or similar tumor types was used. For tumor blood flow and volume fraction of MV, for which parameter values in control (healthy or contralateral) brain tissues are often reported in the same literature, relative values in tumor to control brain were calculated and then multiplied by the representative healthy brain values reported by Saleh *et al.* (13) to calculate the representative tumor parameters. In case of the lack of information, the same values as healthy brain were assumed for parameters below: pH in MV and lysosomes, volume fractions of phospholipids and lysosomes, and ECF bulk flow per brain/tumor volume. Surface area of the tumor BTB was estimated by multiplying that of the healthy brain BBB by the ratio of MV volumes between tumor and healthy brain. The sphere radius of tumor cells was assumed to be equal to that of healthy brain parenchymal cells (13) both in experimental animal models and human patients based on literature information (33, 34).

### Estimation of Correction Factors for Paracellular Permeability Across the Control Brain BBB

Although the LeiCNS-PK3.0 model predicted control brain ECF PK profiles of the six drugs in this study within twofold error for most data points (Supplementary Fig.S1) as reported (13), there was a tendency to overestimate the maximum concentrations (C_max_) of several drugs in control brain ECF and the elimination rate of methotrexate from ECF compartment. The main objective of this study is to adequately describe the impact of pathophysiological alterations on tumor PK profiles and to that end ECF PK profiles in control brain need to be described as accurately as possible, as the “best basis”. By the single parameter sensitivity analyses of pH values, fluid flows, and ECF volume, we found that reduction of paracellular permeability (PPA) across the BBB gave better descriptions of control brain ECF PK profiles for some drugs, while the impact of changing other parameters was negligible. Therefore, we corrected PPA across the control brain BBB to obtain the "best basis" for an appropriate understanding of the impact of pathophysiological alterations on tumor PK. PPA correction factor of each drug in each animal model or patient was estimated by fitting ECF PK data in control brain (healthy, sham or contralateral brain in rat brain tumor models and NE brain region in human patients) to the LeiCNS-PK3.0 model.

### Estimation of Fold Changes of Paracellular Pore Size of the BTB and Active Efflux CL at the BTB Compared with the Control Brain BBB by the “Handshake” Approach

The LeiCNS-PK3.0 model calculates PPA of each drug across the BBB and blood-CSF barrier (BCSFB) based on the aqueous diffusivity coefficient of the drug, the width of the BBB/BCSFB and the surface area of paracellular pore (13, 27). Considering the highly hydrophilic nature of contrast agents used to demonstrate the disruption of the BTB in HGG, it is quite reasonable to attribute their intratumoral accumulation to the increase of their PPA across the BTB compared with the healthy BBB, i.e., opening of paracellular pore of the BTB (7, 8). However, literature information on paracellular pore size of the BTB is very limited and reported values largely vary between literature for animal brain tumor models (35–37), whereas no quantitative information is available for human brain tumor patients. In addition, no quantitative information is available on the expression levels or activities of active transporters at the BTB in animal brain tumor models. For human patients, recent studies using liquid chromatography with tandem mass spectrometry (LC–MS/MS) -based quantitative targeted proteomics demonstrated significant reduction of ABCB1 and ABCG2 protein abundances in isolated MV of GBM and brain metastases compared with non-cancerous cerebral cortex (38, 39). Taken together, pathophysiological information on paracellular pore size of the BTB and active transporter functions at the BTB, both of which are essential in predicting tumor ECF PK profiles by our model, is too limited to perform the “bottom-up” model development, especially for animal brain tumor models. To address the scarce pathophysiological information, we selected to estimate these two parameters of the BTB by fitting existing data to the LeiCNS-tumor model integrated with other available parameters, like a “handshake” with one hand (existing PK data) and the other hand (the model) settling into the best place. Both parameters, as fold changes from those in the control brain BBB, were simultaneously estimated.

### Model Evaluation

Model performance was evaluated by the comparison of predicted PK profiles with the measured ones in plasma, control brain ECF, and brain tumor ECF, where the median and 95% prediction interval of 200 model simulations were compared to *in vivo* measured unbound PK profiles. The model simulations accounted for IIV and RUV of the plasma PK model, as described above. The relevant η of IIV and ε of RUV were randomly sampled from a normal distribution with a mean of 0 and a variance of ω^2^ and σ^2^, respectively, and transformed as required.

Next, prediction errors were calculated using the individual measured drug concentrations and their corresponding time-matched simulated median. Relative accuracy error of a given drug (RA_drug_) at a given compartment was calculated as follows:

$${RA}_{drug}=\frac{1}{M}{\sum }_{i=1}^{N}{\sum }_{j=1}^{m}{log}_{10}\left(\frac{{MedP}_{i,j}}{{Obs}_{i,j}}\right)$$

$$M={\sum }_{i=1}^{N}m$$

where Obs_i,j_ is j^th^ observation of the i^th^ individual; MedP_i,j_ is the median value of the 200 simulations corresponding to Obs_i,j_; M is the total number of observations of all individuals; m is the number of observations of the i^th^ individual; and N is the total number of individuals.

## Results

### Empirical Plasma PK Models

**Fig. 2:**
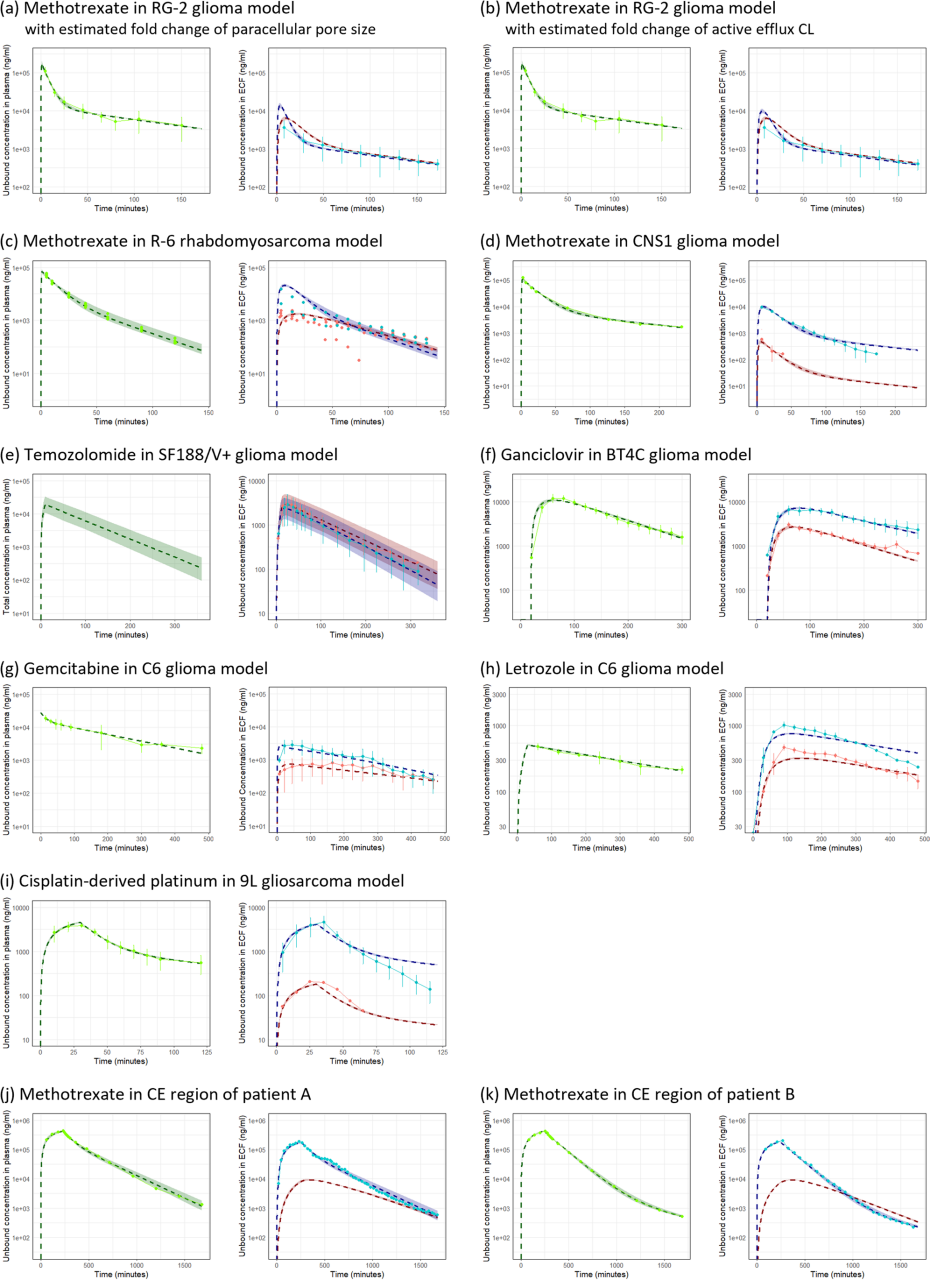
Visual predictive checks plots compared *in vivo* measured drug concentration (dots and solid line; mean ± standard deviation) in plasma (green), control brain (red), and brain tumor (blue) to the median (dashed line) and 95% prediction intervals (colored band) of 200 model simulations. Methotrexate in (**a**) RG-2 glioma model with estimated fold change of paracellular pore size and (**b**) active efflux CL, (**c**) R-6 rhabdomyosarcoma model and (**d**) CNS1 glioma model; (**e**) temozolomide in SF188/V + glioma model; (**f**) ganciclovir in BT4C glioma model; (**g**) gemcitabine in C6 glioma model; (**h**) letrozole in C6 glioma model; (**i**) cisplatin-derived platinum in 9L gliosarcoma model; methotrexate in (**j**) patient A and (**k**) patient B. CE: contrast-enhancing, ECF: brain extracellular fluid.

The empirical plasma PK parameters in rat brain tumor models and human brain tumor patients are listed in Table III. The PK models accurately described the observed unbound PK profiles in plasma as shown in Fig. 2 (green lines and dots). For ganciclovir, introduction of a lag time clearly improved the description of its plasma PK profile, especially C_max_ and the time taken to reach C_max_ (T_max_). Plasma PK model of total temozolomide in SF188/V + glioma model rats was available from the literature (20).

**Table III:** Rat and Human Empirical Plasma PK Models of Unbound Drugs used as Input to the LeiCNS-Tumor Model

Species	Drug	PK data reference	Plasma PK parameter estimates				Interindividual variability		Residual unexplained variability	
			CL_cen_ (mL min^−1^)	Q_cen-per_ (mL min^−1^)	V_cen_ (mL)	V_per_ (mL)	CL_cen_ (%)	V_cen_ (%)	Proportional (%)	Additive (ng mL^−1^)
Rat brain tumor models	Methotrexate	(17)	13.3	10.6	277	661	-	-	8.99	0
		(18)	20.3	4.23	203	80.9	5.67	0	6.14	54.5
		(15)	4.00	1.52	109	163	-	-	10.7	0
	Gemcitabine	(23)	1.81	5.75	225	140	-	-	0	39.2
	Letrozole	(24)	7.61	0	3788	0	-	-	1.58	30.6
	Cisplatin	(16)	3.11	2.57	91.2	183	-	-	5.84	0
			k_el_ (min^−1^)	V_cen_ (mL)	-	-	k_el_ (%)	V_cen_ (%)	Proportional (%)	Additive (ng mL^−1^)
	Temozolomide^a^	(20)	0.0129	233	-	-	6.57	29.3	0	0
			CL_cen_ (mL min^−1^)	V_cen_ (mL)	k_a_ (min^−1^)	Lag time (min)	CL_cen_ (%)	V_cen_ (%)	Proportional (%)	Additive (ng mL^−1^)
	Ganciclovir	(22)	3.59	75.9	0.00924	19.3	-	-	9.14	0
Human brain tumor patients			CL_cen_ (mL min^−1^)	Q_cen-per_ (mL min^−1^)	V_cen_ (mL)	V_per_ (mL)	CL_cen_ (%)	V_cen_ (%)	Proportional (%)	Additive (ng mL^−1^)
	Methotrexate^b^	(25)	163 (A) 174 (B) 214 (C) 160 (D)	63.2 (A) 2.78 (B) 15.1 (C) 157 (D)	20,900 (A) 27,700 (B) 21,800 (C) 25,500 (D)	10,400 (A) 1670 (B) 3870 (C) 7570 (D)	-	-	10.7 (A) 3.85 (B) 13.2 (C) 2.86 (D)	0 (A) 0 (B) 0 (C) 45.9 (D)

CL_cen_: clearance from the central compartment; k_a_: absorption rate constant; k_el_: elimination rate constant from the central compartment; Q_cen-per_: distribution clearance between central and peripheral compartments; V_cen_: central distribution volume; V_per_: peripheral distribution volume

^a^ PK parameters of total temozolomide, reported in (20)

^b^ Parentheses represent patient ID

### Pathophysiological Parameters of Brain Tumors

**Fig. 3:**
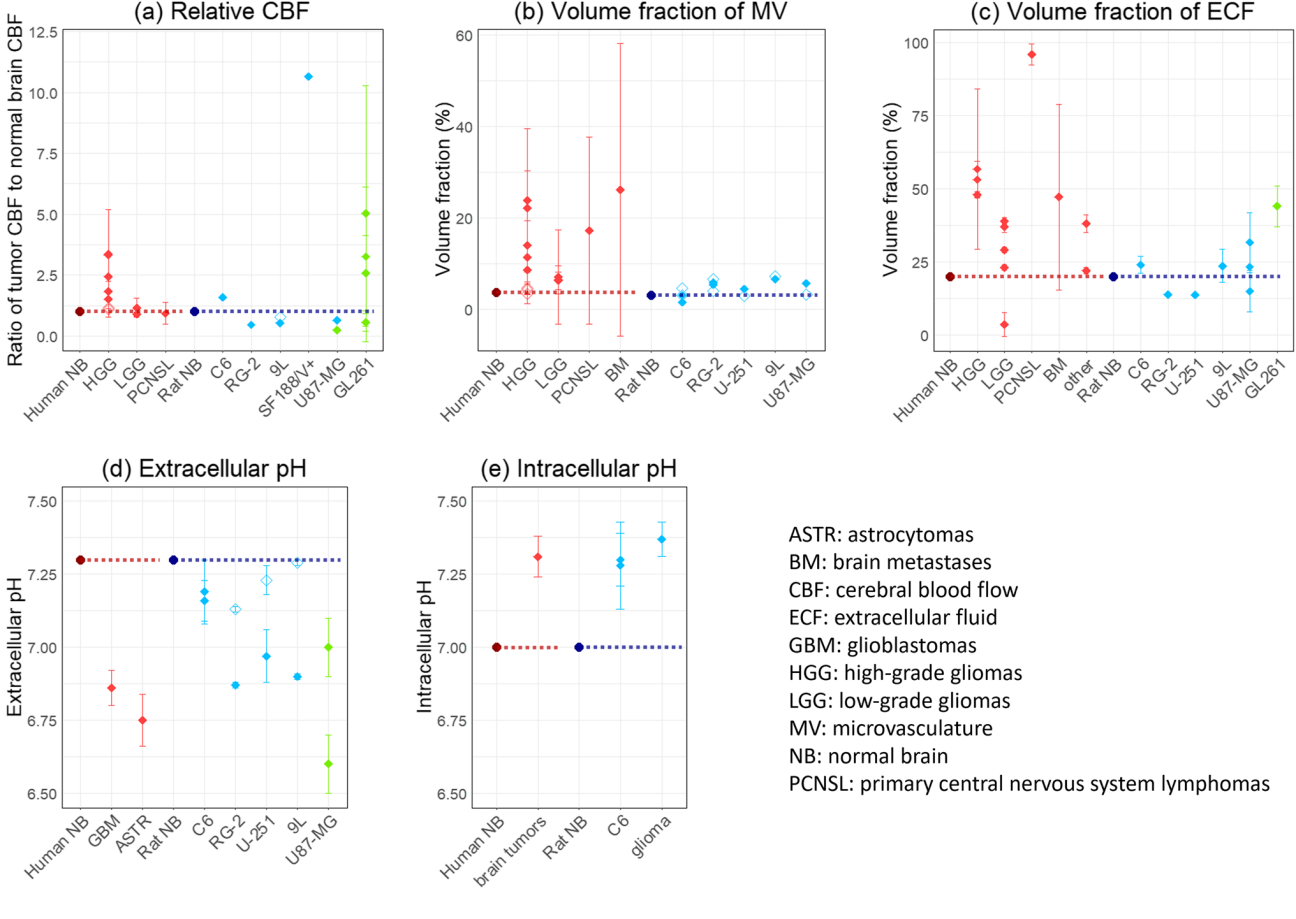
Summary of reported pathophysiological parameters in brain tumors: (**a**) relative CBF to healthy brain, (**b**) volume fraction of MV, (**c**) volume fraction of ECF, (**d**) extracellular pH and (**e**) intracellular pH, including tumor models and tumor types that are not analyzed in this study. Each point represents mean ± standard deviation or standard error of the mean in each literature. Values in human, rat, mouse are shown in red, cyan, and light green, respectively. Closed symbols represent tumor core (animal tumor models) or contrast-enhancing region (human patients), whereas open symbols represent peritumoral region or non-contrast-enhancing region. Dashed lines represent values of healthy brains. References: (a) (13, 40–51); (b) (13, 36, 43, 47, 48, 52–59); (c) (13, 34, 52, 55, 57, 60–64); (d) (13, 65–69); (e) (13, 65, 67, 70).

Five pathophysiological parameters: tumor blood flow, volume fractions of MV and ECF, and extracellular and intracellular pH, in animal brain tumor models and human brain tumors were collected from literature and are summarized in Fig. 3 including parameter values in animal models and tumor types that are not analyzed in this study. Parameter values applied to tumor compartments in this study are summarized in Table IV. Tumor/cerebral blood flow and volume fractions of MV and ECF were all higher in contrast-enhancing (CE) region of HGG than healthy human brain, low-grade gliomas (LGG), and NE region of HGG. In contrast, those values in experimental animal brain tumor models largely varied between models. Interestingly, lower extracellular pH and higher intracellular pH were consistent in all human brain tumors and tumor core of animal brain tumor models.

**Table IV:** Pathophysiological Parameters in Animal Brain Tumor Models and High-Grade Glioma Patients

Species	Tumor model/patient ID (25)	Tumor type	Tumor volume (mL)	Volume fractions (%)		pH		CBF (mL min^−1^ g^−1^)
				MV	ECF	ECF	ICF	
Rat	Healthy brain (13)	-	-	3.00	20.0	7.30	7.00	1.53
Rat brain tumor models	C6	Rat glioma	0.03 (15)	2.25 (52, 53)	24.0 (71)	7.16 (65)	7.28 (65)	2.43 (40)
	BT4C	Rat glioma	0.23 (41)	1.67 (54)	19.0^a^	7.02^a^	7.33^b^	1.14 (41)
	RG-2	Rat glioma	0.075 (32)	5.58 (33, 52)	13.9 (71)	6.87 (67)	7.37^c^	0.698 (42)
	CNS1	Rat glioma	0.03 (15)	3.92^a^	19.0^a^	7.02^a^	7.33^b^	1.56^a^
	R-6	Rat rhabdomyosarcoma	0.17 (29)	6.52^d^	23.6^d^	6.90^d^	7.37^d^	0.819^d^
	9L	Rat gliosarcoma	0.20 (43)	6.52 (43)	23.6 (60)	6.90 (67)	7.37^c^	0.819 (43)
	U87-MG	Human glioblastoma	-	5.61 (55, 56)	23.3 (55, 61)	6.80 (68)	-	0.672 (44, 45)
	SF188/V +	Human glioma	0.12 (20)	20.0^f^	20.9^e^	6.86^e^	7.33^b^	16.27 (46)
	U251	Human glioblastoma	-	4.40 (61)	13.7 (62)	6.97 (69)	-	-
Human	Healthy brain (13)	-	-	3.67	20.0	7.30	7.00	0.527
Human brain tumor patients	A	Glioblastoma	50 (20)	14.9 (47, 48, 57, 58)	54.9 (57, 63)	6.86 (66)	7.31^g^	1.31 (47–50)
	B	Anaplastic astrocytoma	50 (20)	15.5 (47, 57, 58)	52.6 (57, 63, 64)	6.75 (66)	7.31^g^	1.17 (47, 49, 50)
	C, D	Anaplastic oligodendroglioma	50 (20)	16.2 (47, 57)	44.3 (57, 63, 64)	6.81^h^	7.31^g^	1.17 (47, 49, 50)

CBF: cerebral blood flow; ECF: brain extracellular fluid; ICF: brain intracellular fluid; MV: microvasculature

Parentheses represent references

^a^ Average values of RG-2 and C6 (rat gliomas)

^b^ Average values of C6 and “rat glioma”

^c^ Value of “rat glioma” (67)

^d^ Values of 9L (rat gliosarcoma)

^e^ Average values of U251 and U87-MG (human gliomas)

^f^ 4 × average value of U251 and U87-MG (human gliomas) considering its highly vascularized property (MV density in subcutaneous xenograft of SF188/V + glioma was 4 times higher than parental SF188/V- glioma (72))

^g^ Value of “brain tumour” (66)

^h^ Average value of glioblastoma and anaplastic astrocytoma

### Estimation of Correction Factors for PPA Across the Control Brain BBB

Estimated PPA correction factor of each drug is listed in Table V and simulated *versus* observed ECF PK profiles in control brain after PPA correction are shown in Fig. 2 and Supplementary Fig.S1 (red dashed lines and red dots, respectively). Reduction of PPA improved the description of control brain ECF PK profiles, especially C_max_ and T_max_, except for letrozole and cisplatin. These PPA correction factors were applied to the control brain model to obtain the “best basis” for further analyses on tumor compartments except for letrozole and cisplatin, for which no PPA reduction was applied (correction factor = 1). Analysis of ECF PK data of methotrexate in NE region of patient D (25) indicated that no active efflux transport at the BBB is involved and drug elimination from ECF compartment mostly relies on ECF bulk flow (Supplementary Table S1), indicating the BBB function in NE region of patient D may be altered already. Therefore, we selected PK data in NE region of patient C as control brain data and estimated PPA correction factor in patient C was applied to obtain the “best basis” for further analyses of CE regions in patients A and B.

**Table V:** Summary of the Control Brain Models used as the “Best Basis” for the Analyses on Tumor Compartments

Species	Drug	PK data reference	Control brain used as the “best basis”	PPA correction factor^a^ (fold-decrease)	AF_in,ECF_^b^	AF_out,ECF_^b^
Rat brain tumor models	Methotrexate	(17)	Sham brain	14.0	1	4.18E + 06
		(18)	Healthy brain	36.9	1	1.50E + 06
		(15)	Contralateral hemisphere	49.8	1	2.87E + 07
	Temozolomide	(20)	Contralateral hemisphere	5.54	1	166
	Ganciclovir	(22)	Contralateral hemisphere	4.45	1	518
	Gemcitabine	(23)	Healthy brain	80.1	1	74.2
	Letrozole	(24)	Healthy brain	0.573^c^	1^d^	1.30^d^
	Cisplatin	(16)	Contralateral hemisphere	3.70^c^	1^d^	5.93E + 04^d^
Human brain tumor patients	Methotrexate	(25)	Non-contrast-enhancing region	892^e^	1^e^	8.35E + 04^e^

AF: asymmetry factors; ECF: brain extracellular fluid; Kp_uu,ECF_: the ratio of the unbound drug concentration in ECF to that in plasma at steady state; PPA: paracellular permeability

^a^ Estimated by fitting the observed ECF PK data in control brain to the LeiCNS-PK3.0 model and applied to the LeiCNS-tumor model to obtain the “best basis” for further analyses on tumor compartments

^b^ Calculated from control brain Kp_uu,ECF_ using the LeiCNS-PK3.0 equations at steady state as previously reported (13)

^c^ No correction factor was applied to the control brain model due to the negligible impact on PK profile

^d^ Without PPA correction factor

^e^ Values in patient C

### Estimation of Fold Changes of Paracellular Pore Size of the BTB and Active Efflux CL at the BTB Compared with the Control Brain BBB

Estimated fold changes of paracellular pore size of the BTB and active efflux CL at the BTB over the control brain BBB of each drug in each animal brain tumor model or HGG patient are listed in Table VI. Estimated fold change of paracellular pore size ranged from 0.172 to 49.1, whereas that of active efflux CL ranged from -1.17 to 7.81 in animal models. In human patients, fold changes of both parameters were estimated to be higher than 1. For methotrexate in RG-2 glioma model (17), each parameter was separately estimated with the other fixed to 1 because simultaneous estimation of both parameters resulted in varied estimates depending on initial parameters. This is because the same fold changes of paracellular pore size (increase) and active BTB/BBB efflux CL (decrease) have very similar impact on PK profile, and thus parameter sets with the same ratio of these fold changes gave almost the same PK profiles. For letrozole in the C6 glioma model (24), estimated fold change of active efflux CL was below 0, indicating the involvement of active influx transport as well as disappeared active efflux transport at the BTB. Out of 9 cases for six small molecule drugs tested in this study, the increase of paracellular pore size of the BTB compared with the BBB was estimated in 8 cases except for temozolomide in the SF188/V + glioma model (20), whereas the decrease of active efflux CL was estimated in 7 cases except for gemcitabine in the C6 glioma model (23) and methotrexate in human HGG patients (25).

**Table VI:** Fold Changes of Paracellular Pore Size and Active Efflux Clearance in the Brain Tumor BTB Over the Control Brain BBB Estimated by the “Handshake” Approach

Species	Drug	PK data reference	Tumor model	Fold changes in the brain tumor BTB over the control brain BBB^a^	
				Paracellular pore size	Active efflux clearance
Rat brain tumor models	Methotrexate	(17)	RG-2	2.66^b^	0.392^b^
		(18)	R-6	17.2	0.804
		(15)	CNS1	7.46	0.131
	Temozolomide	(20)	SF188/V +	0.172	0.269
	Ganciclovir	(22)	BT4C	2.50	0.101
	Gemcitabine	(23)	C6	21.8	7.81
	Letrozole	(24)	C6	49.1	-1.17^c^
	Cisplatin	(16)	9L	8.12	0.0295
Human brain tumor patients	Methotrexate^d^	(25)	-	76.0 (A) 2210 (B)	2.62 (A) 54.7 (B)

^a^ Simultaneously estimated by the “handshake” approach, i.e., fitting the observed ECF PK data in brain tumor to the LeiCNS-tumor model integrated with other available pathophysiological parameters shown in Table IV

^b^ Separately estimated with the other parameter fixed to 1

^c^ Indicates the involvement of active influx transport as well as the completely diminished active efflux transport

^d^ Parentheses represent patient ID

### Model Evaluation

Simulated tumor ECF PK profiles using estimated fold changes of paracellular pore size of the BTB and active efflux CL at the BTB over the control brain BBB are shown in Fig. 2 (blue dashed lines) in comparison to the observed PK profiles (blue dots). The relative accuracy errors are shown in Supplementary Fig.S2. The LeiCNS-tumor model well captured ECF PK profiles in brain tumor as well as control brain within twofold error for most data points, except for methotrexate in the R-6 rhabdomyosarcoma model (29). In this case, IIV for plasma PK parameters were not large enough to explain the interindividual difference in the observed ECF PK profiles, indicating the involvement of IIV for other factors in CNS physiology accounting for interindividual differences in brain tumor and control brain ECF PK profiles.

## Discussion

Tumor recurrence in GBM patients is inevitable (3). The limited drug exposure within tumor and brain around tumor (BAT) remains a major issue limiting the long-term efficacies of anticancer agents for GBM (73). Micrometastatic tumor cells, which cause tumor recurrence, are often undetectable and unresectable, and protected by the intact BBB (3, 8), whereas CE region with dense tumor has the disrupted BTB (7). Such heterogeneous properties of tumor MV, not only in terms of permeability but also perfusion, are ones of the rate-limiting factors in effective therapy of malignant brain tumors (8). Therefore, the drug candidates selected for clinical trials for malignant brain tumors should be those that are able to penetrate the intact BBB as well as the disrupted BTB. Ideally, prediction of ECF PK profiles in three (or more) different compartments; (A) tumor with the disrupted BTB, (B) tumor with the intact BBB, and (C) healthy brain with the intact BBB, would be the best approach to address the tumor heterogeneity. Due to the very limited information on pathophysiological alterations in (B) tumor with the intact BBB, however, firstly we aimed to build the LeiCNS-tumor model focusing on (A) tumor with the disrupted BTB and (C) normal-appearing brain with the intact BBB in this study. If the LeiCNS-tumor model successfully predicts ECF PK profiles in both (A) and (C), it can be further extended to (B) and more complex heterogenous tumors when the pathophysiological information becomes available. Furthermore, some of the pathophysiological parameters of brain tumors are quite scarce even for (A) tumor with the disrupted BTB. The development of novel therapeutic agents for malignant brain tumors is an urgent matter and every possible effort to improve the possibility of success in the drug development should be made immediately, instead of waiting until enough information become available. Therefore, in this study, we used the “handshake” approach to understand and extract pathophysiological alterations in brain tumors from existing data on tumor PK.

It is generally accepted that it is the unbound drug that equilibrates over biological membranes and is able to interact with target molecules, and MD is a key technique to obtain time-dependent information on unbound drug concentration in ECF of the target tissues (74). Although intratumoral MD has been performed also in neuro-oncology, clinical examples are quite rare due to ethical restrictions of human brain sampling (75, 76). In addition, its labor-intensiveness and requirement of high technical skills make it difficult to routinely perform MD experiments during the drug discovery process even in animal brain tumor models, especially in parallel with efficacy studies. The ultimate goal of this research is to establish a comprehensive brain tumor PBPK model that can be used to predict unbound PK profiles in human patients to support the successful drug development for malignant brain tumors. Nevertheless, it must be of importance to gain a quantitative understanding of the impact of the pathophysiological alterations on unbound PK profiles in experimental animal tumor models, for the right selection of clinical candidates and efficiently translating the understanding to human patients in clinical trials. From these points of view, application of PBPK modeling in predicting unbound PK profiles in brain tumor tissues is of great significance not only in human patients but also in animal brain tumor models.

The LeiCNS-PK3.0 model simulations demonstrated that alteration of physiological processes in CNS diseases, especially of paracellular pore size, pH of ECF and ICF, can affect both rate and extent of passive drug transport across the BBB (14). This result motivated us to extend the LeiCNS-PK3.0 model for the prediction of PK profiles specifically in brain tumors, in combination with information on altered pathophysiological properties in brain tumors. Quantitative information on pathophysiological alterations under disease conditions is the most important requirement in predicting PK profiles in the relevant population.

Physical and biological tumor microenvironment can contribute to the tumor progression, invasion, maintenance of the stem-like fraction and treatment resistance (77–79) as well as the altered drug disposition (25, 80). For example, aggressive angiogenesis induced by brain tumors during its progression results in structurally and functionally abnormal blood vessels, leading to alterations of blood flow, volume fraction of MV, and BTB/BBB function compared with healthy brain (8, 81). Also, large amounts of protons and lactate produced by aerobic glycolysis in tumor cells are then transported to extracellular space by membrane transporters (70), which creates reversed pH gradient across cell membrane with more acidic ECF and more basic ICF than healthy brain (65, 66). As shown in Fig. 3, blood flow and volume fractions of MV and ECF were higher in CE region of HGG than healthy human brain, LGG, and NE region of HGG, whereas those values in experimental animal brain tumor models were model-dependent, indicating that conditions of their MV differ among models and between preclinical models and clinical settings. These differences may be one of the reasons of discrepancy between drug efficacies in preclinical and clinical, especially in terms of drug delivery to the target tumor tissues. In contrast, lower extracellular pH and higher intracellular pH than healthy brain were consistent between animal brain tumor models and human brain tumors. As demonstrated in the previous simulations (14), altered pH can have a large impact on the rate and extent of the BBB transport of acidic or basic drugs. For example, Kp_uu,ECF_ of methotrexate in RG-2 glioma was equivalent to or slightly lower than that in sham rat brain (Table I, (17)), which may mistakenly lead to the idea that there is no significant alteration of BTB functions in this glioma model compared with the healthy BBB. Methotrexate is acidic and thus is less ionized in more acidic tumor ECF than healthy brain ECF. Therefore, unbound methotrexate concentrations in ECF should be lower in tumor than healthy brain, assuming the same BTB/BBB functions and that only unionized molecule can pass through the BTB/BBB via transcellular pathway. In our model analysis including pH information, altered BTB function in RG-2 glioma model was actually estimated (2.66-fold increase of paracellular pore size or 2.55-fold decrease of active efflux CL over control (sham) brain BBB, Table VI) even though Kp_uu,ECF_ are almost the same. This result is a good example showing the significance of considering pH differences in interpreting and analyzing PK data in multiple tissues or under different (patho-) physiological conditions.

We unexpectedly found that reduction of PPA improved the description of PK profiles of some drugs in control brain ECF (Table IV, Supplementary Fig.S1 and Fig. 2). It should be noted again that the LeiCNS-PK3.0 model prediction of control brain ECF PK profiles without PPA correction was acceptable (within twofold error for most data points, see Supplementary Fig.S1) as our previous study (13) and no PPA correction was required for letrozole and cisplatin. Accordingly, we believe the utility of the LeiCNS-PK3.0 model as the healthy brain model has not been changed by the findings in this study. Considering that all drugs in this study but letrozole are highly hydrophilic (logP_o/w_ < 0) whereas only limited number of hydrophilic drugs were included in the original study (13), however, these findings indicate that the LeiCNS-PK3.0 model may overestimate passive transport rate across the BBB of highly hydrophilic drugs whose major transport route is estimated to be the paracellular pathway. It is worth noting that most drug candidates targeting CNS are generally not highly hydrophilic. Nevertheless, we consider these findings indicating an improvement opportunity of the LeiCNS-PK3.0 model. Possible explanations of PPA overestimation by the LeiCNS-PK3.0 model in this study include charge effect, size effect, tortuosity, and study conditions. Firstly, PPA of negatively charged molecules across Caco-2 monolayer and several BMEC models was reported to be about half of uncharged molecule with similar molecular size, due to the negatively charged residues lining the paracellular pores (82, 83). Secondly, at least a part of paracellular pores are size-restricted, where PPA can be described by the Renkin hydrodynamic sieving function (83, 84). These effects are probably the main reasons why estimated PPA correction factors were higher for methotrexate than those for other drugs except for gemcitabine. Thirdly, tortuosity of paracellular pathway needs to be considered (82). These three factors are currently not specified in the LeiCNS-PK3.0 model and should be addressed in future research. In addition, it is also possible that study conditions including animal strain, experimental apparatus, and MD probe location, make inter-laboratory differences in the BBB condition. The LeiCNS-PK3.0 model has an advantage in capturing many details of general CNS physiology at the species level, but physiology may be different within species (i.e., between rat strains). We expect that expanding the validation dataset will allow to include above factors and further improve the estimation of PPA based on the physicochemical properties.

Alterations of paracellular pore size (35–37) and expression levels of active efflux transporters (38, 85) in the BTB in animal glioma models or human GBM patients from the healthy brain BBB are reported. However, quantitative information on these two parameters are currently very limited compared with other pathophysiological parameters, even though they are essential for the PK prediction with the LeiCNS-tumor model. Instead, we estimated both parameters by fitting existing PK data in tumor ECF to our model integrated with other available pathophysiological parameters. We believe this is the best possible approach to address the lack of quantitative information and quite useful to learn about what could happen in disease conditions for which so little quantitative data is available. First, the increase of paracellular pore size of the tumor BTB compared with the control brain BBB was estimated in 8 out of 9 cases including human patients (Table VI), which is consistent with general belief of leakier MV in brain tumors (5, 6). The only exception was temozolomide in the SF188/V + glioma model rats. Since temozolomide is highly hydrophilic (logP_o/w_ = -1.153), paracellular route is estimated to account for > 99.6% of its passive transport across the BBB by the LeiCNS-PK3.0 model. Therefore, the increase of paracellular pore size of the BTB should lead to the increase of PPA across the BTB and tumor ECF exposure, whereas existing data show its ECF exposure in tumor is slightly lower than in contralateral brain (Table I, (20)). Considering many reports describing temozolomide as a lipophilic drug (86–88), it may be able to efficiently permeate a lipid bilayer somehow, despite its extremely low logP_o/w_. Indeed, Dr. Avdeef estimated that transcellular route is the major passive transport pathway of temozolomide (89). This is also a significant subject for future studies to improve the prediction of transport rate across the BBB. The range of fold change of BTB paracellular pore size was between 0.172 and 2210, which indicates model- and condition-dependent alteration of the BTB integrity in brain tumors. The fold change of 2210 (paracellular pore size of 1547 nm) for methotrexate in patient B was exceptionally high, nevertheless, it is comparable to the mean BTB paracellular pore size in RG-2 glioma model rats, 1.1 µm (36). Other cases are consistent with the reported range of upper limit of BTB paracellular pore size, 7–100 nm (37). In addition, the current analysis suggests that opening of paracellular pore of the BTB in brain tumors is common between animal models and human patients, while only one human case (two patients) was available in this study. We expect expanding data set will allow to examine the possibility of quantitative extrapolation directly from animal models to human patients regarding the paracellular pore size of the BTB in brain tumors.

Next, the decrease of active efflux CL at the BTB compared with the control brain BBB was estimated in 7 out of 9 cases (Table VI), which is consistent with recent publication reporting significant reduction of ABCB1 and ABCG2 protein abundances in isolated MV of GBM in human patients compared with non-cancerous cerebral cortex (38). Although no quantitative information is available for other transporters and animal brain tumor models, similar pathophysiological alterations would be highly possible. Importantly, protein abundances of all efflux transporters measured (ABCB1, ABCG2, and ABCC4) in MV of GBM were below the lower limit of quantification in some patients (38), which is consistent with the disappeared active efflux transport of letrozole estimated in the current analysis. Two exceptional cases are gemcitabine in the C6 glioma model rats and methotrexate in human HGG patients. Efflux transporters for gemcitabine include P-gp, MRP1, MRP5, and MRP7 (Supplementary Table S2). Considering that active efflux transport of letrozole, a weak P-gp substrate (90), was estimated to be completely diminished at the BTB in the same C6 glioma model, upregulation of MRPs might explain this exceptional case. However, it is difficult to precisely interpret this result with limited information. In human patients, MRP5 protein was abundantly detected in the BMEC of almost all glioma samples, whereas MRP1 was not detected on the protein level (91). Methotrexate is recognized by quite various transporters (Supplementary Table S2), but a series of transporter knockout mice studies (92–96) indicates that BCRP and MRP4 mainly contribute to the active efflux of methotrexate at the BBB at least in mice. Considering that median BCRP protein level in MV of GBM was reduced to 35% of normal levels whereas MRP4 was undetectable in both normal human brain and GBM (38), the present estimate of higher active efflux CL at the BTB in HGG than that at the control brain BBB seems inappropriate. This discrepancy may be explained by the level of active efflux transport function at the BBB in control brain used as the “best basis” in this analysis. We selected PK data in NE brain region of patient C as the “best basis” for an appropriate understanding of the impact of pathophysiological alterations in HGG because the model analysis indicated that active efflux transport was completely disappeared in NE region of patient D (Supplementary Table S1). However, it is highly possible that active efflux transport function was already decreased also in patient C. In fact, extrapolation of human active efflux CL from rat data based on the relative expression factor approach (97, 98) largely underestimated the ECF exposure of methotrexate in patient C (data not shown). Furthermore, similar finding has recently been reported by Li *et al.* (99), where reduction of ABCB1 abundance compared with the non-cancerous brain BBB was required to describe unbound PK profile of ribociclib in NE brain region of GBM patients. Although a lack of accumulation of contrast agents in NE brain regions indicates an intact BBB function in terms of limited PPA by tight junctions, micrometastatic tumor cells may possibly exist there (3, 8, 9) and affect the efflux transporter functions, possibly more drastically than in CE region.

The LeiCNS-tumor model predicted tumor ECF PK profiles within twofold error for most data points (Fig. 2), in combination with estimated fold changes of paracellular pore size of the BTB and active efflux CL at the BTB over the control brain BBB. Although further validation with more drugs is required, this result suggest that the LeiCNS-tumor model can be used to predict tumor ECF PK for which pathophysiological parameters are available. Importantly, five pathophysiological parameters (blood flow, volume fractions of MV and ECF, and extracellular/intracellular pH) in brain tumors are available from literature for various brain tumor models and tumor types (Fig. 3). Accordingly, it is possible to predict tumor ECF PK depending on the tumor models, tumor types and tumor grades. The lack of two pathophysiological parameters disables the fully “bottom-up” model building and instead we estimated them by fitting existing data in this study. These estimates represent the BTB function of each animal tumor model and human patient analyzed in this study, i.e., model- and patient-specific parameters. Considering the different estimates in the same C6 glioma model rats for gemcitabine and letrozole, careful consideration of small changes of experimental conditions (e.g., days after inoculation of tumor cells) is required. Nevertheless, once the model-specific estimates of these two parameters are obtained for each animal brain tumor model, the LeiCNS-tumor model can be used to predict tumor ECF PK profiles of other drugs in the same animal model using the model-specific estimates, available pathophysiological parameters, Kp_uu,ECF_ in healthy brain and physicochemical properties of drugs of interest. Accordingly, we believe our model has a potential to improve the efficiency of drug discovery process for malignant brain tumors, through enhancing the efficient understanding of PKPD relationships of brain tumors as well as the right selection of drug candidates for clinical trials.

All drugs but temozolomide in this study showed higher ECF exposure in brain tumor than in control brain. Other molecules including contrast agents (7, 9), ribociclib (99), paclitaxel (100, 101), and Texas Red (101) also demonstrated higher exposure in brain tumors. Although these results are consistent with the generally believed leakier MV in brain tumors (5, 6), they are inconsistent with the poor success rate of clinical candidates for malignant brain tumors and may be unintuitive. As stated above, micrometastatic tumor cells protected by the intact BBB can cause tumor recurrence, which is one of the significant reasons of difficulty in treating malignant brain tumors. In addition, it is important to note that higher exposure in brain tumor tissue does not necessarily expect desired anti-tumor efficacy and therefore an appropriate understanding of PKPD relationship is necessary for the successful drug development for malignant brain tumors.

There are several limitations in our new LeiCNS-tumor model. First, some parameters in tumor compartments were assumed to be the same as healthy brain due to the lack of quantitative information available. In particular, ECF bulk flow may be different between brain tumor and healthy brain, considering that tumor has an increased interstitial fluid pressure and the dense extracellular matrix (77, 81, 102). Recent progress of magnetic resonance imaging (MRI) technique is expected to enable the measurement of tumor ECF bulk flow (103). Second, only a limited number of drugs are evaluated in the present study. More drugs, with distinctively different physicochemical properties, are needed to be included in the future analyses. Third, no spatial information is considered in the present model. Concentrations of drugs which penetrate the disrupted BTB more efficiently than the intact BBB are likely to be highest in the tumor core and decrease with distance from the core, in the order of tumor periphery, tumor edge-brain interface, BAT, and healthy brain distant from tumor (100, 101). Dividing the tumor model into several regional tumor models including heterogenous MV properties will be quite informative and a relevant subject for future research. Moreover, a 3D modeling approach (104–106) in combination with spatial information on pathophysiology is expected to allow the prediction of local distribution of drugs and biomarkers. Finally, PBPK model analyses depend on the availability of quantitative information on (patho-) physiology. As far as we know, there is no quantitative information on paracellular pore size of the BTB and only limited information on expression levels of active transporters at the BTB in brain tumor patients. In order to effectively utilize the LeiCNS-tumor model for improving the possibility of success in drug development for malignant brain tumors through predicting PK profiles in brain tumors, more quantitative information on tumor pathophysiological alterations, especially paracellular pore size of the BTB and expression levels of active transporters at the BTB, is desired. Although estimation of these parameters based on MD data in human patients may be difficult as demonstrated in this study for methotrexate, fitting MRI data with contrast agents to our model will allow to estimate heterogenous paracellular pore size of the BTB in human patients. Furthermore, considering that MRI with contrast agents is a standard diagnosis of brain tumors (7), this approach will be able to give not only representative parameters (e.g., mean) in the relevant population but also individual parameters which may allow the “tailor-made” PK prediction in each patient. In addition, quantitative information on the expression levels of active transporters in tumor MV by LC–MS/MS-based targeted proteomics (38, 39), instead of parameter estimation by fitting, will further enhance the predictability and utility of the LeiCNS-tumor model.

## Conclusion

In conclusion, we extended the LeiCNS-PK3.0 model specifically for the prediction of brain tumor ECF PK profiles by adding brain tumor compartments and integrating pathophysiological parameters of brain tumors. The LeiCNS-tumor model was able to describe the ECF PK profiles in brain tumor as well as control brain in combination with estimated paracellular pore size of the BTB and active efflux CL at the brain tumor BTB. Although further research is required, the current results demonstrated its potential to contribute to the efficient drug discovery and development for malignant brain tumors.

## Supplementary Information

Below is the link to the electronic supplementary material.

Supplementary file1 (PDF 590 KB)

## Data Availability

Data generated during and analyzed during the current study are not publicly available due privacy but are available from the corresponding author on reasonable request.
