# Supplementary material for: The Extension of the LeiCNS-PK3.0 Model in Combination with the “Handshake” Approach to Understand Brain Tumor Pathophysiology
Source: Pharm Res. 2022 Mar 7;39(7):1343–61. doi: 10.1007/s11095-021-03154-1 (PMC9246813; doi:10.1007/s11095-021-03154-1)
Supplement: Supplementary file 1 — Supplementary file1 (PDF 590 KB) [file 11095_2021_3154_MOESM1_ESM.pdf]

Supplementary material

The Extension of the LeiCNS-PK3.0 Model in Combination with the “Handshake” Approach to Understand Brain Tumor Pathophysiology

Makoto Hirasawa[1,*], Mohammed A. A. Saleh[1], Elizabeth C. M. de Lange[1]

[1] Division of Systems Biomedicine and Pharmacology, Leiden Academic Centre for Drug Research, Leiden University, Leiden, The Netherlands

*Corresponding author
Mailing address: Einsteinweg 55, 2333 CC, Leiden, The Netherlands
Telephone: +31 71 527 6330
E-mail address: m.hirasawa@lacdr.leidenuniv.nl

Supplementary Table S1: Estimated clearance values of methotrexate in non-contrast-enhancing region of high-grade glioma patients

Patient ID	PPA correction factor	$AF_{out,ECF}$	Clearance (mL min^{-1})			
			Passive transcellular efflux	Passive paracellular efflux	Active efflux	ECF bulk flow
C	892	8.35E+04	5.19E-06	0.0371	0.546	0.202
D	1087	< 0	5.19E-06	0.0304	< 0	0.202

AF: asymmetry factors, ECF: brain extracellular fluid, PPA: paracellular permeability

Supplementary Table S2: Transporters involved in the transport of each drug

Drug	Transporters
Methotrexate	P-gp[1-3], BCRP[4-6], MRP1[7,8], MRP2[8,9], MRP3[7,10], MRP4[6,11], MRP8[12], OAT1[13,14], OAT2[15,16], OAT3[13,14,17], OAT4[13], OATP1B1[18,19], OATP1B3[19], OATP1C1[20], OATP3A1[21], OATP4C1[22], PEPT1[23], PAT1[23], PCFT[24], RFC1[25], FOLR1[23,26], FOLR2[23,26]
Temozolomide	P-gp[27-31], BCRP[30-32]
Ganciclovir	P-gp[33], OAT1[34], OCT1[34,35], MATE1[36,37], MATE2-K[36,37]
Gemcitabine	P-gp[38,39], MRP1[39], MRP5[39], MRP7[40], ENT1[41-44], ENT2[41], CNT1[41], CNT3[41,44]
Letrozole	P-gp[45]
Cisplatin	OCT2[46,47], MRP2[48], MRP6[48,49], CTR1[50], CTR2[51], ATP7A[52], ATP7B[52,53]

Parentheses represent reference numbers

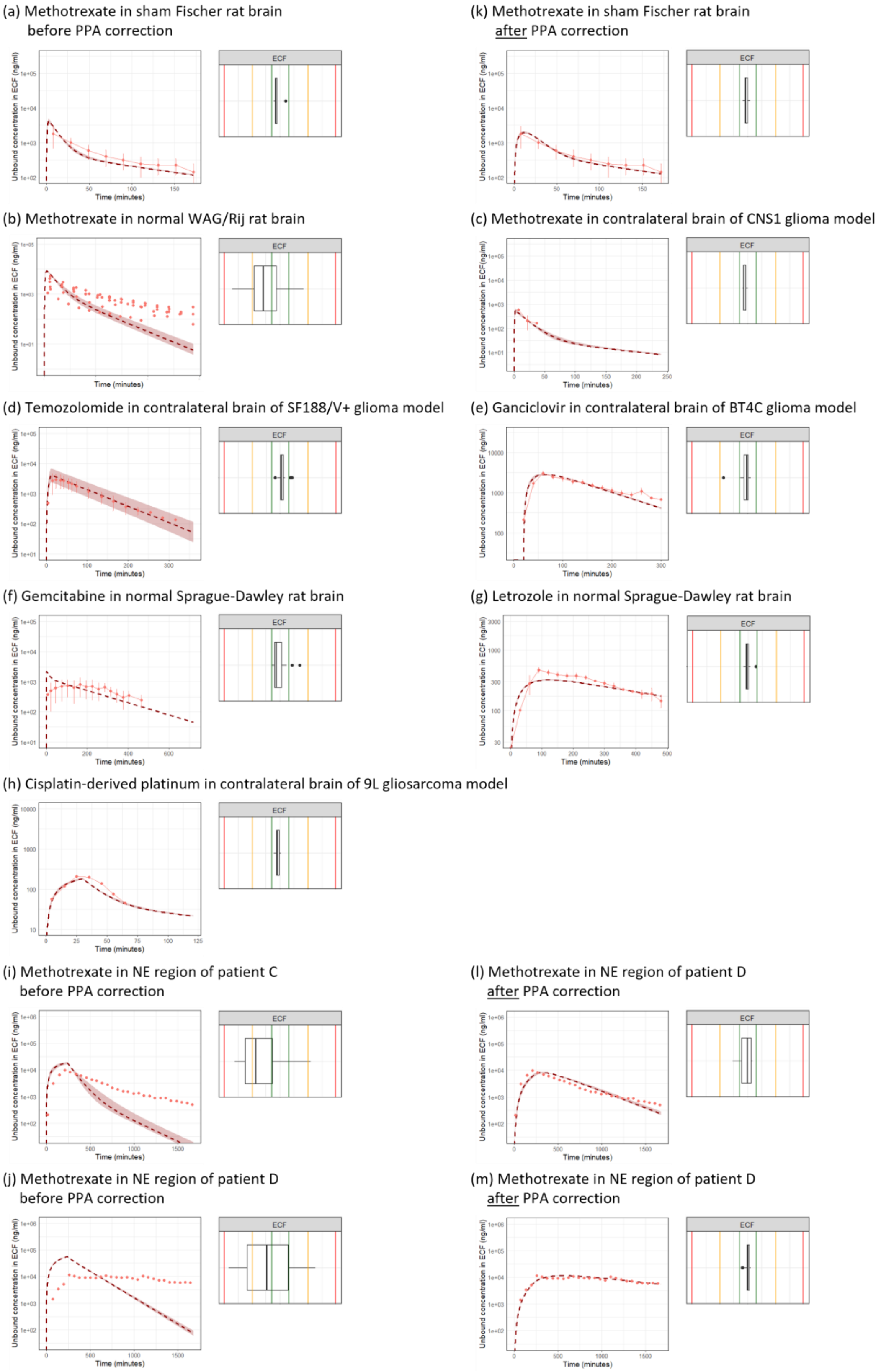

Supplementary Fig.S1 Simulated versus observed ECF PK profiles and boxplots of the relative accuracy error in control brain before correction of the PPA. Green and yellow solid lines in boxplots represent two- and ten- fold error, respectively. Methotrexate in (a) sham Fischer rat brain, (b) normal WAG/Rij rat brain, (c) contralateral hemisphere of CNS1 glioma model; (d) temozolomide in contralateral hemisphere of SF188/V+ glioma model; (e) ganciclovir in contralateral hemisphere of BT4C glioma model; (f) gemcitabine in normal Sprague-Dawley rat brain; (g) letrozole in normal Sprague-Dawley rat brain; (h) cisplatin-derived platinum in contralateral hemisphere of 9L gliosarcoma model; methotrexate in (i) NE region of patient C and (j) patient D. Those after correction of the PPA of methotrexate in (k) sham Fischer rat brain and (l) NE region of patient C and (m) patient D. ECF: brain extracellular fluid, NE: non-contrast-enhancing, PPA: paracellular permeability

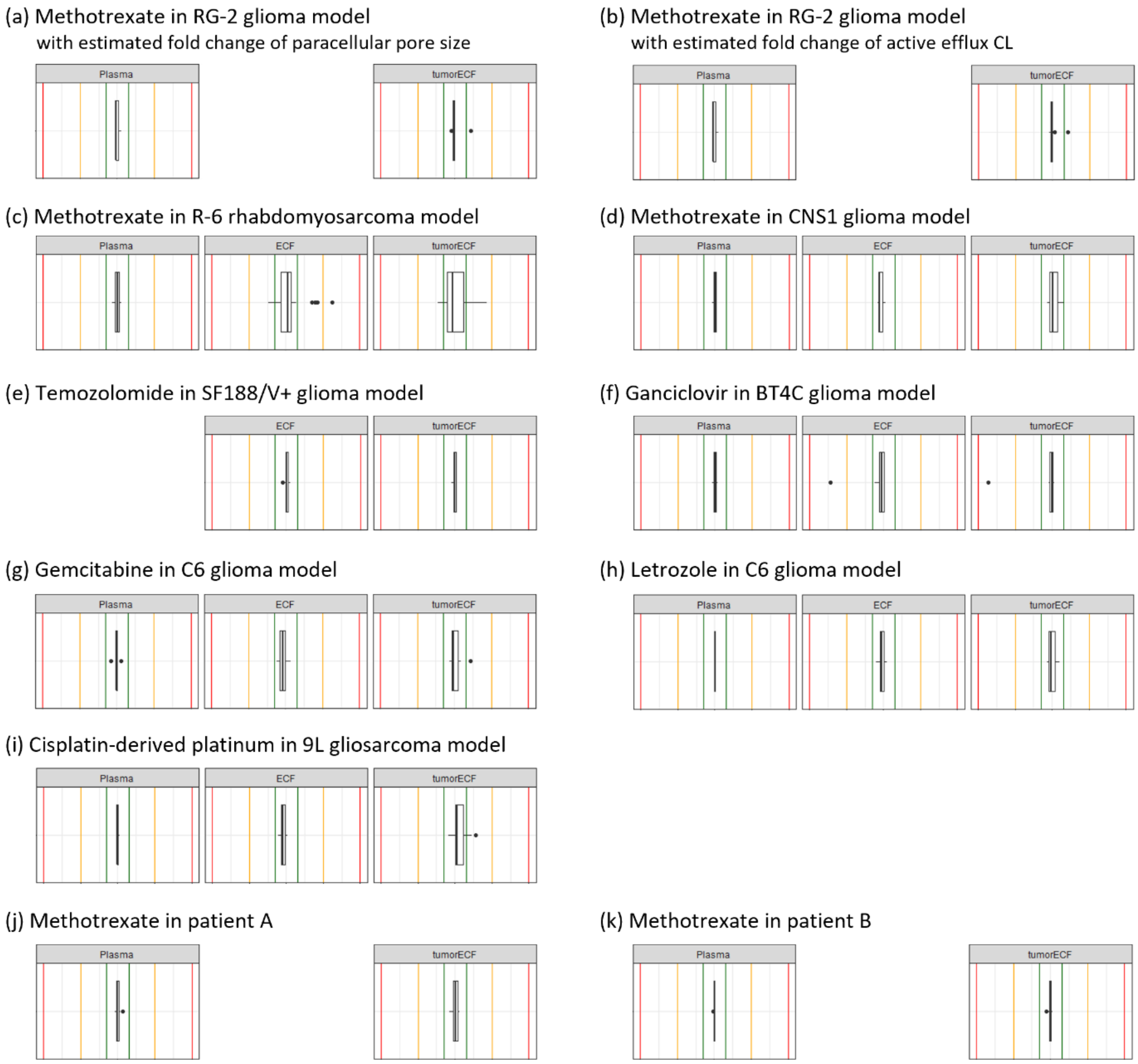

Supplementary Fig.S2 Boxplots of the relative accuracy error. Green and yellow solid lines represent two- and ten- fold error, respectively. Methotrexate in (a) RG-2 glioma model with estimated fold change of paracellular pore size and (b) active efflux CL, (c) R-6 rhabdomyosarcoma model and (d) CNS1 glioma model; (e) temozolomide in SF188/V+ glioma model; (f) ganciclovir in BT4C glioma model; (g) gemcitabine in C6 glioma model; (h) letrozole in C6 glioma model; (i) cisplatin-derived platinum in 9L gliosarcoma model; methotrexate in (j) patient A and (k) patient B. CL: clearance, ECF: brain extracellular fluid

References

1. de Graaf D, Sharma RC, Mechetner EB, Schimke RT, Roninson IB. P-glycoprotein confers methotrexate resistance in 3T6 cells with deficient carrier-mediated methotrexate uptake. Proc Natl Acad Sci U S A. 1996;93(3):1238-42. https://doi.org/10.1073/pnas.93.3.1238.
2. Norris MD, De Graaf D, Haber M, Kavallaris M, Madafiglio J, Gilbert J, et al. Involvement of MDR1 P-glycoprotein in multifactorial resistance to methotrexate. Int J Cancer. 1996;65(5):613-9. https://doi.org/10.1002/(SICI)1097-0215(19960301)65:5<613::AID-IJC10>3.0.CO;2-8.
3. Ogushi N, Sasaki K, Shimoda M. CAN a P-gp modulator assist in the control of methotrexate concentrations in the rat brain? -inhibitory effects of rhodamine 123, a specific substrate for P-gp, on methotrexate excretion from the rat brain and its optimal route of administration. J Vet Med Sci. 2017;79(2):320-327. https://doi.org/10.1292/jvms.16-0315.
4. Chen ZS, Robey RW, Belinsky MG, Shchaveleva I, Ren XQ, Sugimoto Y, et al. Transport of methotrexate, methotrexate polyglutamates, and 17beta-estradiol 17-(beta-D-glucuronide) by ABCG2: effects of acquired mutations at R482 on methotrexate transport. Cancer Res. 2003;63(14):4048-54.
5. Vlaming ML, Pala Z, van Esch A, Wagenaar E, de Waart DR, van de Wetering K, et al. Functionally overlapping roles of Abcg2 (Bcrp1) and Abcc2 (Mrp2) in the elimination of methotrexate and its main toxic metabolite 7-hydroxymethotrexate in vivo. Clin Cancer Res. 2009;15(9):3084-93. https://doi.org/10.1158/1078-0432.CCR-08-2940.
6. Sane R, Wu SP, Zhang R, Gallo JM. The effect of ABCG2 and ABCC4 on the pharmacokinetics of methotrexate in the brain. Drug Metab Dispos. 2014;42(4):537-40. https://doi.org/10.1124/dmd.113.055228.
7. Zeng H, Chen ZS, Belinsky MG, Rea PA, Kruh GD. Transport of methotrexate (MTX) and folates by multidrug resistance protein (MRP) 3 and MRP1: effect of polyglutamylation on MTX transport. Cancer Res. 2001;61(19):7225-32.
8. Bakos E, Evers R, Sinkó E, Váradi A, Borst P, Sarkadi B. Interactions of the human multidrug resistance proteins MRP1 and MRP2 with organic anions. Mol Pharmacol. 2000;57(4):760-8. https://doi.org/10.1124/mol.57.4.760.
9. Li L, Agarwal S, Elmquist WF. Brain efflux index to investigate the influence of active efflux on brain distribution of pemetrexed and methotrexate. Drug Metab Dispos. 2013;41(3):659-67. https://doi.org/10.1124/dmd.112.049254.
10. Akita H, Suzuki H, Hirohashi T, Takikawa H, Sugiyama Y. Transport activity of human MRP3 expressed in Sf9 cells: comparative studies with rat MRP3. Pharm Res. 2002;19(1):34-41. https://doi.org/10.1023/a:1013699130991.
11. Kanamitsu K, Kusuhara H, Schuetz JD, Takeuchi K, Sugiyama Y. Investigation of the Importance of Multidrug Resistance-Associated Protein 4 (Mrp4/Abcc4) in the Active Efflux of Anionic Drugs Across the Blood-Brain Barrier. J Pharm Sci. 2017;106(9):2566-2575. https://doi.org/10.1016/j.xphs.2017.04.040.
12. Chen ZS, Guo Y, Belinsky MG, Kotova E, Kruh GD. Transport of bile acids, sulfated steroids, estradiol 17-beta-D-glucuronide, and leukotriene C4 by human multidrug resistance protein 8 (ABCC11). Mol Pharmacol. 2005;67(2):545-57. https://doi.org/10.1124/mol.104.007138.
13. Takeda M, Khamdang S, Narikawa S, Kimura H, Hosoyamada M, Cha SH, et al. Characterization of methotrexate transport and its drug interactions with human organic anion transporters. J Pharmacol Exp Ther. 2002;302(2):666-71. https://doi.org/10.1124/jpet.102.034330.
14. Uwai Y, Iwamoto K. Transport of aminopterin by human organic anion transporters hOAT1 and hOAT3: Comparison with methotrexate. Drug Metab Pharmacokinet. 2010;25(2):163-9. https://doi.org/10.2133/dmpk.25.163.
15. Sekine T, Cha SH, Tsuda M, Apiwattanakul N, Nakajima N, Kanai Y, et al. Identification of multispecific organic anion transporter 2 expressed predominantly in the liver. FEBS Lett. 1998;429(2):179-82. https://doi.org/10.1016/s0014-5793(98)00585-7.
16. Sun W, Wu RR, van Poelje PD, Erion MD. Isolation of a family of organic anion transporters from human liver and kidney. Biochem Biophys Res Commun. 2001;283(2):417-22. https://doi.org/10.1006/bbrc.2001.4774.

17. VanWert AL, Sweet DH. Impaired clearance of methotrexate in organic anion transporter 3 (Slc22a8) knockout mice: a gender specific impact of reduced folates. Pharm Res. 2008;25(2):453-62. https://doi.org/10.1007/s11095-007-9407-0..
18. van de Steeg E, van der Kruijssen CM, Wagenaar E, Burggraaff JE, Mesman E, Kenworthy KE, et al. Methotrexate pharmacokinetics in transgenic mice with liver-specific expression of human organic anion-transporting polypeptide 1B1 (SLCO1B1). Drug Metab Dispos. 2009;37(2):277-81. https://doi.org/10.1124/dmd.108.024315.
19. Abe T, Unno M, Onogawa T, Tokui T, Kondo TN, Nakagomi R, et al. LST-2, a human liver-specific organic anion transporter, determines methotrexate sensitivity in gastrointestinal cancers. Gastroenterology. 2001;120(7):1689-99. https://doi.org/10.1053/gast.2001.24804.
20. Pizzagalli F, Hagenbuch B, Stieger B, Klenk U, Folkers G, Meier PJ. Identification of a novel human organic anion transporting polypeptide as a high affinity thyroxine transporter. Mol Endocrinol. 2002;16(10):2283-96. https://doi.org/10.1210/me.2001-0309.
21. Adachi H, Suzuki T, Abe M, Asano N, Mizutamari H, Tanemoto M, et al. Molecular characterization of human and rat organic anion transporter OATP-D. Am J Physiol Renal Physiol. 2003;285(6):F1188-97. https://doi.org/10.1152/ajprenal.00402.2002.
22. Mikkaichi T, Suzuki T, Onogawa T, Tanemoto M, Mizutamari H, Okada M, et al. Isolation and characterization of a digoxin transporter and its rat homologue expressed in the kidney. Proc Natl Acad Sci U S A. 2004;101(10):3569-74. https://doi.org/10.1073/pnas.0304987101.
23. Inoue K, Yuasa H. Molecular basis for pharmacokinetics and pharmacodynamics of methotrexate in rheumatoid arthritis therapy. Drug Metab Pharmacokinet. 2014;29(1):12-9. https://doi.org/10.2133/dmpk.dmpk-13-rv-119.
24. Nakai Y, Inoue K, Abe N, Hatakeyama M, Ohta KY, Otagiri M, et al. Functional characterization of human proton-coupled folate transporter/heme carrier protein 1 heterologously expressed in mammalian cells as a folate transporter. J Pharmacol Exp Ther. 2007;322(2):469-76. https://doi.org/10.1124/jpet.107.122606.
25. Qiu A, Jansen M, Sakaris A, Min SH, Chattopadhyay S, Tsai E, et al. Identification of an intestinal folate transporter and the molecular basis for hereditary folate malabsorption. Cell. 2006;127(5):917-28. https://doi.org/10.1016/j.cell.2006.09.041.
26. Sharma S, Das M, Kumar A, Marwaha V, Shankar S, Aneja R, et al. Interaction of genes from influx-metabolism-efflux pathway and their influence on methotrexate efficacy in rheumatoid arthritis patients among Indians. Pharmacogenet Genomics. 2008;18(12):1041-9. https://doi.org/10.1097/fpc.0b013e328311a8fd.
27. Schaich M, Kestel L, Pfirrmann M, Robel K, Illmer T, Kramer M, et al. A MDR1 (ABCB1) gene single nucleotide polymorphism predicts outcome of temozolomide treatment in glioblastoma patients. Ann Oncol. 2009;20(1):175-81. https://doi.org/10.1093/annonc/mdn548.
28. Zhang R, Saito R, Shibahara I, Sugiyama S, Kanamori M, Sonoda Y, et al. Temozolomide reverses doxorubicin resistance by inhibiting P-glycoprotein in malignant glioma cells. J Neurooncol. 2016;126(2):235-42. https://doi.org/10.1007/s11060-015-1968-x.
29. Goldwirt L, Beccaria K, Carpentier A, Farinotti R, Fernandez C. Irinotecan and temozolomide brain distribution: a focus on ABCB1. Cancer Chemother Pharmacol. 2014;74(1):185-93. https://doi.org/10.1007/s00280-014-2490-0.
30. de Gooijer MC, de Vries NA, Buckle T, Buil LCM, Beijnen JH, Boogerd W, et al. Improved Brain Penetration and Antitumor Efficacy of Temozolomide by Inhibition of ABCB1 and ABCG2. Neoplasia. 2018;20(7):710-720. https://doi.org/10.1016/j.neo.2018.05.001.
31. Lin F, de Gooijer MC, Roig EM, Buil LC, Christner SM, Beumer JH, et al. ABCB1, ABCG2, and PTEN determine the response of glioblastoma to temozolomide and ABT-888 therapy. Clin Cancer Res. 2014;20(10):2703-13. https://doi.org/10.1158/1078-0432.CCR-14-0084.
32. Martín V, Sanchez-Sanchez AM, Herrera F, Gomez-Manzano C, Fueyo J, Alvarez-Vega MA, et al. Melatonin-induced methylation of the ABCG2/BCRP promoter as a novel mechanism to overcome multidrug resistance in brain tumour stem cells. Br J Cancer. 2013;108(10):2005-12. https://doi.org/10.1038/bjc.2013.188.
33. Li M, Si L, Pan H, Rabba AK, Yan F, Qiu J, et al. Excipients enhance intestinal absorption of ganciclovir by

P-gp inhibition: assessed in vitro by everted gut sac and in situ by improved intestinal perfusion. Int J Pharm. 2011;403(1-2):37-45. https://doi.org/10.1016/j.ijpharm.2010.10.017.

34. Takeda M, Khamdang S, Narikawa S, Kimura H, Kobayashi Y, Yamamoto T, et al. Human organic anion transporters and human organic cation transporters mediate renal antiviral transport. J Pharmacol Exp Ther. 2002;300(3):918-24. https://doi.org/10.1124/jpet.300.3.918.

35. Wagner DJ, Hu T, Wang J. Polyspecific organic cation transporters and their impact on drug intracellular levels and pharmacodynamics. Pharmacol Res. 2016;111:237-246. https://doi.org/10.1016/j.phrs.2016.06.002.

36. Tanihara Y, Masuda S, Sato T, Katsura T, Ogawa O, Inui K. Substrate specificity of MATE1 and MATE2-K, human multidrug and toxin extrusions/H(+)-organic cation antiporters. Biochem Pharmacol. 2007;74(2):359-71. https://doi.org/10.1016/j.bcp.2007.04.010.

37. Nies AT, Damme K, Schaeffeler E, Schwab M. Multidrug and toxin extrusion proteins as transporters of antimicrobial drugs. Expert Opin Drug Metab Toxicol. 2012;8(12):1565-77. https://doi.org/10.1517/17425255.2012.722996.

38. Bergman AM, Pinedo HM, Talianidis I, Veerman G, Loves WJ, van der Wilt CL, et al. Increased sensitivity to gemcitabine of P-glycoprotein and multidrug resistance-associated protein-overexpressing human cancer cell lines. Br J Cancer. 2003;88(12):1963-70. https://doi.org/10.1038/sj.bjc.6601011.

39. Kohan HG, Boroujerdi M. Time and concentration dependency of P-gp, MRP1 and MRP5 induction in response to gemcitabine uptake in Capan-2 pancreatic cancer cells. Xenobiotica. 2015;45(7):642-52. https://doi.org/10.3109/00498254.2014.1001809.

40. Hopper-Borge E, Xu X, Shen T, Shi Z, Chen ZS, Kruh GD. Human multidrug resistance protein 7 (ABCC10) is a resistance factor for nucleoside analogues and epothilone B. Cancer Res. 2009;69(1):178-84. https://doi.org/10.1158/0008-5472.CAN-08-1420.

41. Mackey JR, Yao SY, Smith KM, Karpinski E, Baldwin SA, Cass CE, et al. Gemcitabine transport in xenopus oocytes expressing recombinant plasma membrane mammalian nucleoside transporters. J Natl Cancer Inst. 1999;91(21):1876-81. https://doi.org/10.1093/jnci/91.21.1876.

42. Morinaga S, Nakamura Y, Watanabe T, Mikayama H, Tamagawa H, Yamamoto N, et al. Immunohistochemical analysis of human equilibrative nucleoside transporter-1 (hENT1) predicts survival in resected pancreatic cancer patients treated with adjuvant gemcitabine monotherapy. Ann Surg Oncol. 2012 ;19 Suppl 3:S558-64. https://doi.org/10.1245/s10434-011-2054-z.

43. Marcé S, Molina-Arcas M, Villamor N, Casado FJ, Campo E, Pastor-Anglada M, et al. Expression of human equilibrative nucleoside transporter 1 (hENT1) and its correlation with gemcitabine uptake and cytotoxicity in mantle cell lymphoma. Haematologica. 2006;91(7):895-902.

44. Govindarajan R, Leung GP, Zhou M, Tse CM, Wang J, Unadkat JD. Facilitated mitochondrial import of antiviral and anticancer nucleoside drugs by human equilibrative nucleoside transporter-3. Am J Physiol Gastrointest Liver Physiol. 2009;296(4):G910-22. https://doi.org/10.1152/ajpgi.90672.2008.

45. Miyajima M, Kusuhara H, Takahashi K, Takashima T, Hosoya T, Watanabe Y, et al. Investigation of the effect of active efflux at the blood-brain barrier on the distribution of nonsteroidal aromatase inhibitors in the central nervous system. J Pharm Sci. 2013;102(9):3309-19. https://doi.org/10.1002/jps.23600.

46. Pan BF, Sweet DH, Pritchard JB, Chen R, Nelson JA. A transfected cell model for the renal toxin transporter, rOCT2. Toxicol Sci. 1999;47(2):181-6. https://doi.org/10.1093/toxsci/47.2.181.

47. Burger H, Zoumaro-Djayoon A, Boersma AW, Helleman J, Berns EM, Mathijssen RH, et al. Differential transport of platinum compounds by the human organic cation transporter hOCT2 (hSLC22A2). Br J Pharmacol. 2010;159(4):898-908. https://doi.org/10.1111/j.1476-5381.2009.00569.x.

48. Sanchez-Covarrubias L, Slosky LM, Thompson BJ, Davis TP, Ronaldson PT. Transporters at CNS barrier sites: obstacles or opportunities for drug delivery? Curr Pharm Des. 2014;20(10):1422-49. https://doi.org/10.2174/13816128113199990463.

49. Belinsky MG, Chen ZS, Shchaveleva I, Zeng H, Kruh GD. Characterization of the drug resistance and transport properties of multidrug resistance protein 6 (MRP6, ABCC6). Cancer Res. 2002;62(21):6172-7.

50. Howell SB, Safaei R, Larson CA, Sailor MJ. Copper transporters and the cellular pharmacology of the platinum-containing cancer drugs. Mol Pharmacol. 2010;77(6):887-94. https://doi.org/10.1124/mol.109.063172.

51. Blair BG, Larson CA, Safaei R, Howell SB. Copper transporter 2 regulates the cellular accumulation and cytotoxicity of Cisplatin and Carboplatin. Clin Cancer Res. 2009;15(13):4312-21. https://doi.org/10.1158/1078-0432.CCR-09-0311.
52. Rabik CA, Maryon EB, Kasza K, Shafer JT, Bartnik CM, Dolan ME. Role of copper transporters in resistance to platinating agents. Cancer Chemother Pharmacol. 2009;64(1):133-42. https://doi.org/10.1007/s00280-008-0860-1.
53. Mangala LS, Zuzel V, Schmandt R, Leshane ES, Halder JB, Armaiz-Pena GN, et al. Therapeutic Targeting of ATP7B in Ovarian Carcinoma. Clin Cancer Res. 2009;15(11):3770-80. https://doi.org/10.1158/1078-0432.CCR-08-2306.
